# Associations of sexually transmitted infections and bacterial vaginosis with abnormal cervical cytology: A cross-sectional survey with 9090 community women in China

**DOI:** 10.1371/journal.pone.0230712

**Published:** 2020-03-26

**Authors:** Wu Li, Lan-lan Liu, Zhen-zhou Luo, Chun-yan Han, Qiu-hong Wu, Li Zhang, Li-shan Tian, Jun Yuan, Tao Zhang, Zhong-wei Chen, Tu-bao Yang, Tie-jian Feng, Min Zhang, Xiang-sheng Chen

**Affiliations:** 1 Shenzhen Nanshan Center for Chronic Disease Control, Shenzhen, China; 2 Shenzhen Nanshan Maternity & Child Healthcare Hospital, Shenzhen, China; 3 Department of Epidemiology and Health Statistics, Xiangya School of Public Health, Central South University, Changsha, Hunan, China; 4 Shenzhen Center for Chronic Disease Control, Shenzhen, China; 5 National Center for STD Control, Nanjing, China; University of Texas Health Science Center at San Antonio, UNITED STATES

## Abstract

**Background:**

Although it is well acknowledged that persistent infection with high-risk human papillomavirus types in genital sites plays a crucial role in the development of squamous cell cervical carcinoma, there is no unanimous consensus on the association between non-HPV sexually transmitted infections and abnormal cervical cytology.

**Methods:**

In the present study, we evaluated cervical cytology status, sexually transmitted infections and bacterial vaginosis status, and collected social-demographic information among recruited participants to explore the association of STIs and bacterial vaginosis with abnormal cervical cytology.

**Results:**

9,090 women’s specimens were successfully tested, with a total of 8,733 (96.1%) women had normal cytology and 357 (3.9%) women exhibited abnormal cytology. The prevalence of HPV, *Chlamydia trachomatis*, *Neisseria gonorrhoeae*, and bacterial vaginosis was significantly higher in the ≥ASC-US group than the NILM group (*P*<0.05). Women with *Neisseria gonorrhoeae* infection (AOR = 5.30, 95% CIs = 1.30–21.51, *P* = 0.020) or bacterial vaginosis (AOR = 1.94, 95% CIs = 1.08–3.47, *P* = 0.026) exhibited an increased risk of abnormal cervical cytology after adjusted for carcinogenic HPV-positive status.

**Conclusions:**

Our results demonstrated that *Neisseria gonorrhoeae* infection in genital sites and/or bacterial vaginosis may independently increase the risk for cervical cytology abnormalities after adjusted for carcinogenic HPV-positive status. Besides, these results improved our understanding of the etiology of abnormal cervical cytology and may be useful for the management of women with ASC-US cytology.

## Background

Based on epidemiological and laboratory evidence, persistent infection with high-risk human papillomavirus (HPV) types in genital sites plays a crucial role in the development of squamous cell carcinoma (SCC) of the cervix [1, 2]. After the initial detection of high-risk HPV infection, the development of SCC of the cervix usually spanned decades and underwent a lengthy stage of squamous intraepithelial lesion (SIL) [2, 3]. Furthermore, most HPV infections are transient and only a small proportion of high-risk HPV infections persists and leads to high-grade squamous intraepithelial lesions or SCC [3], which suggests that other factors may facilitate the occurrence of SIL during this long transition period.

Previous studies reported that there are many other risk factors for SCC including long term use of oral contraceptives [4], high parity [5], cigarette smoking [6], co-infection with the human immunodeficiency virus [7], and sexually transmitted infections (STIs), such as *Chlamydia trachomatis* [8], *Neisseria gonorrhoeae* [9], herpes simplex virus type 2 [10, 11], *Trichomonas vaginalis* [12], *Mycoplasma* [13], *Ureaplasma* species [14, 15]. However, at present, there is no unanimous consensus on the impact of non-HPV STIs on abnormal cervical cytology. For example, as the most prevalent STI worldwide, *Chlamydia trachomatis* infection is considered as a risk factor for abnormal cervical cytology [16] or cervical intraepithelial neoplasia 2 (CIN-2) [17], but, Safaeian et al. [18] found no positive associations between *Chlamydia trachomatis* infection and cervical pre-malignancy after controlled for carcinogenic HPV-positive status.

Bacterial vaginosis relates to a remarkable shift in the vaginal microbiota to a dysbiotic state, marked by a diverseness of microorganism and increased loads of aerotolerant and strict anaerobes, including *Gardnerella vaginalis*, *Mobiluncus* and *Aptopobium vaginae*, and other fastidious bacteria such as *Megasphaera*, *Sneathia*, and *Clostridiales spp* [19]. Previous studies revealed that bacterial vaginosis is not only associated with reproductive and obstetric sequelae [20], but also with cervical pre-cancerous lesions [21]. Nevertheless, more studies are needed to verify the association and consolidate that evidence [17].

Hence, we investigated the association of STIs and bacterial vaginosis with abnormal cervical cytology among community women, which would improve our knowledge about the etiology of SCC and may be useful for disease control and prevention.

## Methods

### Study procedure and specimen collection

Our previous study [22] has described the study area and sample source in detail. From March to August 2017, we recruited participants from 9,249 women who met eligibility criteria and provided informed consent in our previous study [22], and all of the women signed an informed consent to this study. Women who met any of the following exclusion criteria were not enrolled: pregnancy, without a history of sexual activity, sexual intercourse three days ago, menstrual period, previous hysterectomy, vaginal bleeding, vaginal douching or using a vaginal suppository, currently suffering from gynecological inflammation. The inclusion criteria were the same as inclusion criteria in our previous study [22]: being a female resident, aged 20–60 years and living locally in Shenzhen city Nanshan District during the past 3 months. All participants signed informed consent and were interviewed using a structured questionnaire to collect socio-demographic and clinical information before enrollment. All participants voluntarily agreed to provide a self-administrated 3–5 mL first-catch urine specimen (*Chlamydia trachomatis* and *Neisseria gonorrhoeae* tests), a cervical swab (HPV tests), two vaginal swabs (gynecological examinations), and an exfoliated cervical cells specimen (liquid-based cervical cytology test).

After allocating a unique identification number to each participant, two research nurses were assigned to check the integrity of questionnaire information and instruct participants in urine specimen collection, and guide participants to a specialized room for specimen collection. After collecting participant’s specimens, laboratory testing procedures were started immediately. Laboratory staffs were blinded to the clinical findings.

### *Chlamydia trachomatis* and *Neisseria gonorrhoeae* DNA test

The method and procedures of *Chlamydia trachomatis* and *Neisseria gonorrhoeae* DNA tests were described in our previous study [22].

### HPV DNA test

A skilled gynecologist collected the cervical swab for HPV tests. Swabs were taken and placed into a tube containing 3 mL cell preservation medium, and were stored at -20°C until testing. Fourteen high-risk HPV genotypes (16, 18, 31, 33, 35, 39, 45, 51, 52, 56, 58, 59, 66, 68) and two low-risk HPV types (6, 11) in all cervical swabs were detected by using the PCR-based MALDI-TOF-MS assays [23, 24] in the Center of BGI Health clinical laboratory (BGI, Shenzhen, China). GBI Shenzhen designed the MALDI-TOF-MS-based HPV multiplex assay and HPV genotyping assay with excellent specificity [24]. China Food and Drug Administration approved the performance of the PCR-based multiplex genotyping and sequencing assays.

### Gynecological examination

Vaginal secretions swab specimens were collected by two skilled gynecologists and were rolled on to a glass slide for Gram staining immediately. Vaginal cleanliness, detection of hyphae and spores of *Candidiasis* and clue cells were confirmed by Gram staining of vaginal secretions. *Trichomonas Vaginalis* was diagnosed by microscopic examination of wet mounts immediately once the vaginal secretions swab collected. Amine test, pH of vaginal secretions and leukocytes were further confirmed within fifteen minutes. The diagnosis of bacterial vaginosis was based on Amsel’s criteria [25], which was widely adopted to the clinical diagnosis of bacterial vaginosis. A positive diagnosis of bacterial vaginosis was made once three of the four following signs are present: the presence of clue cells, an adherent and homogenous grayish-white vaginal discharge, a vaginal pH exceeding a value of 4.5, a fishy or amine odor after the addition of a 10% potassium hydroxide solution.

### Liquid-based cytology test

Exfoliated cervical cells specimens were collected by two gynecologists with conical cytobrush and were placed into a tube with 4 mL preservation solution and were temporarily stored at 4°C condition until the required test. Cervical cytology samples were diagnosed by using liquid-based cytology technique (TriPath Imaging Inc., Burlington, USA) according to the manufacturer’s instruction and were doubled-checked by cytotechnologists without information on STIs test results. The 2001 Bethesda system [26] was employed to classify the reported cytological results as following: negative for intraepithelial lesion or malignancy (NILM); Epithelial cell abnormalities including atypical squamous cells of undetermined significance (ASC-US), atypical squamous cells that cannot exclude HSIL (ASC-H), low-grade squamous intraepithelial lesion (LSIL), high-grade squamous intraepithelial lesion (HSIL), and atypical glandular cells (AGC). All abnormal epithelial cell specimens, as well as a randomly selected sample of normal cytology, were referred for a colposcopic examination and, if necessary, biopsy.

### Statistical analysis

Anonymous data collection and statistical analysis were performed by Microsoft Excel 2016 and R-statistics software (version 3.4.1) respectively. Abnormal cytology group (≥ASC-US group) was defined as women who had a diagnosis of the following cytology findings: ASC-US, ASC-H, LSIL, HSIL or AGC. Variables were evaluated for comparisons between normal cytology (only NILM finding) group and ≥ASC-US group (include all abnormal cytological findings) or ASC-US group (only ASC-US finding) or LSIL+HSIL group (only LSIL finding and only HSIL finding) for analytical calculations. Prevalence of STIs and bacterial vaginosis were presented as positive rate and corresponding 95% confidential intervals (95% CIs). Chi-square test (χ^2^), or two-sided Fisher exact test for 2×2 contingency table was used to evaluate the statistical significance of rates of variables between different groups. A univariate analysis was employed to estimate the associations of variables with different cytological findings. Variables with a significance level of *p*<0.2 were included in the multivariate logistic regression model adjusted by potential confounders. Crude odds ratios (OR), Adjusted odds ratio (AOR) and corresponding 95% CIs were calculated. A *p*<0.05 was considered significant. All statistical analyses were performed using SPSS 19.0 statistical software (SPSS lnc. Chicago, IL) and R-statistics 3.4.3 software.

### Ethics approval and consent to participate

The Ethical Review Committee of the Shenzhen Nanshan Center for Chronic Disease Control reviewed and approved the study (Approval No. LL20170017). A written informed consent was obtained from all participants. All experiments and procedures were performed in accordance with relevant guidelines and regulations of the People’s Republic of China. Participants who tested positive for STIs or abnormal cervical cytology were contacted privately by a nurse for further treatment and other interventions.

## Results

### Overall prevalence

Out of the 9,249 women who met eligibility criteria and signed an informed consent, 156 (1.69%) women were excluded because of they met exclusion criteria, 9,093 (98.3%) women completed the questionnaire interview and provided all specimens, 3 urine specimens were invalid for *Chlamydia trachomatis* DNA and *Neisseria gonorrhoeae* DNA and failed to re-sample. 9,090 (98.3%) women’s specimens were successfully tested, a total of 8,733 (96.1%) women had normal cytology (NILM) and 357 (3.9%) women exhibited abnormal cytology: 181 ASC-US (2.0%), 16 ASC-H (0.2%), 115 LSIL (1.3%), 40 HSIL (0.4%), 5 AGC (0.06%). High-risk HPV infection (6.77%, 95% CIs: 6.25%-7.29%) had the highest prevalence among detected STIs. The prevalence of bacterial vaginosis and *Trichomonas vaginalis* was 2.27% (95% CIs: 1.96%-2.58%) and 0.35% (95% CIs: 0.23%-0.47%), respectively (**Table 1**).

**Table 1:** Prevalence of STIs and bacterial vaginosis among participants.

Variables ^*a*^	Test Results	No.	Prevalence (95% CIs)*
High-risk human papillomavirus	Negative	8475	6.77% (6.25%-7.29%)
	Positive	615	
*Chlamydia trachomatis*	Negative	8715	4.12% (3.71%-4.53%)
	Positive	375	
*Neisseria gonorrhoeae*	Negative	9074	0.18% (0.09%-0.27%)
	Positive	16	
*Trichomonas vaginalis*	Negative	9058	0.35% (0.23%-0.47%)
	Positive	32	
Vulvovaginal candidiasis	Negative	8561	5.82% (5.34%-6.30%)
	Positive	529	
Bacterial vaginosis	Negative	8884	2.27% (1.96%-2.58%)
	Positive	206	

^*a*^ Positive refers to women not only positive for corresponding variables but also may co-infection with other sexually transmitted pathogens as well and/or positive for bacterial vaginosis at the same time

* 95% CIs, 95% confidence intervals; *P* value <0.05 was considered significant.

### Analysis of prevalent HPV genotype and cytology status

As shown in **Table 2**, HPV-52 (1.8%) was the most prevalent genotype in studied population, followed by HPV-16 (1.1%), HPV-58 (1.0%), HPV-18 (0.5%). Among 357 women exhibited abnormal cytology, 192 women positive for high-risk HPV, with a prevalence of 52.1% (186/357, 95% CIs: 46.92%-57.28%). HPV-52 (12.9%) was the most prevalent genotype in ≥ASC-US group, followed by HPV-58 (10.4%), HPV-16 (8.4%), and HPV-51 (5.0%). The prevalence of high-risk HPV genotypes in abnormal groups (≥ASC-US group: except for HPV-35, HPV-45; ASC-US group: except for HPV-18, HPV-31, HPV-56, HPV-66; LSIL+HSIL group: except for HPV-68) were significantly higher than NILM group (**Table 2**). No cases of AGC were positive for HPV.

**Table 2:** The prevalence of HPV genotypes by cytology status.

HPV infection	Overall (n = 9090)	NILM (n = 8733)	≥ASC−US (n = 357)			ASC−US (n = 181)			LSIL+HSIL (n = 155)		
	n (%)	n (%)	n (%)	*P* value*	OR (95% CIs)**	n (%)	*P* value*	OR (95% CIs)**	n (%)	*P* value*	OR (95% CIs)**
HPV−6	19 (0.2)	14 (0.2)	5 (1.4)	**<0.001**	8.8 (3.2–24.7)	4 (2.2)	**<0.001**	14.1 (4.6–43.2)	1 (0.7)	0.178	4.0 (0.5–30.9)
HPV−11	5 (0.1)	4 (0.1)	1 (0.3)	0.105	6.1 (0.7–55.0)	1 (0.6)	**0.026**	12.1 (1.3–109.0)	0	—	—
HPV−16	100 (1.1)	70 (0.8)	30 (8.4)	**<0.001**	11.4 (7.3–17.7)	4 (2.2)	**0.048**	2.8 (1.0–7.7)	21 (13.6)	**<0.001**	19.4 (11.6–32.5)
HPV−18	47 (0.5)	39 (0.5)	8 (2.2)	**<0.001**	5.1 (2.4–11.0)	2 (1.1)	0.211	2.5 (0.6–10.4)	6 (3.9)	**<0.001**	9.0 (3.7–21.5)
HPV−31	21 (0.2)	13 (0.2)	8 (2.2)	**<0.001**	15.4 (6.3–37.3)	1 (0.6)	0.206	3.7 (0.5–28.6)	6 (3.9)	**<0.001**	27 (10.1–72.0)
HPV−33	25 (0.3)	16 (0.2)	9 (2.5)	**<0.001**	14.1 (6.2–32.1)	2 (1.1)	**0.017**	6.1 (1.4–26.7)	6 (3.9)	**<0.001**	21.9 (8.5–56.8)
HPV−35	13 (0.1)	11 (0.1)	2 (0.6)	0.052	4.5 (1.0–20.2)	0	—	—	2 (1.3)	**0.002**	10.4 (2.3–47.2)
HPV−39	36 (0.4)	27 (0.3)	9 (2.5)	**<0.001**	8.3 (3.9–17.9)	3 (1.7)	**0.006**	5.4 (1.6–18.1)	6 (3.9)	**<0.001**	13.0 (5.3–31.9)
HPV−45	14 (0.2)	12 (0.1)	2 (0.6)	0.066	4.1 (0.9–18.4)	0	—	—	2 (1.3)	**0.003**	9.5 (2.1–42.8)
HPV−51	40 (0.4)	22 (0.3)	18 (5.0)	**<0.001**	21.0 (11.2–39.6)	8 (4.4)	**<0.001**	18.3 (8.0–41.7)	10 (6.5)	**<0.001**	27.3 (12.7–58.7)
HPV−52	163 (1.8)	117 (1.3)	46 (12.9)	**<0.001**	10.9 (7.6–15.6)	20 (11.1)	**<0.001**	9.1 (5.6–15.1)	23 (14.8)	**<0.001**	12.8 (7.9–20.7)
HPV−56	17 (0.2)	11 (0.1)	6 (1.7)	**<0.001**	13.6 (5.0–36.9)	1 (0.6)	0.157	4.4 (0.6–34.3)	5 (3.2)	**<0.001**	26.4 (9.1–77.0)
HPV−58	86 (1.0)	49 (0.6)	37 (10.4)	**<0.001**	20.5 (13.2–31.9)	6 (3.3)	**<0.001**	6.1 (2.6–14.4)	28 (18.1)	**<0.001**	39.1 (23.8–64.2)
HPV−59	11 (0.1)	9 (0.1)	2 (0.6)	**0.030**	5.5 (1.2–25.4)	0	—	—	2 (1.3)	**<0.001**	12.7 (2.7–59.1)
HPV−66	25 (0.3)	13 (0.2)	12 (3.4)	**<0.001**	23.3 (10.6–51.5)	1 (0.6)	0.206	3.7 (0.5–28.6)	11 (7.1)	**<0.001**	51.2 (22.6–116.3)
HPV−68	45 (0.5)	36 (0.4)	9 (2.5)	**<0.001**	6.2 (3.0–13.1)	7 (3.9)	**<0.001**	9.7 (4.3–22.1)	2 (1.3)	0.116	3.2 (0.8–13.2)
HPV	638 (7.0)	446 (5.1)	192 (53.8)	**<0.001**	21.6 (17.2–27.2)	58 (32.0)	**<0.001**	8.8 (6.3–12.1)	122 (78.7)	**<0.001**	68.7 (46.2–102.1)
High-risk HPV	616 (6.8)	430 (4.9)	186 (52.1)	**<0.001**	21 (16.7–26.4)	53 (29.3)	**<0.001**	8.0 (5.7–11.2)	121 (78.1)	**<0.001**	68.7 (46.4–101.8)
Single HPV	608 (6.7)	428 (4.9)	180 (50.4)	**<0.001**	19.7 (15.7–24.8)	56 (30.9)	**<0.001**	8.7 (6.3–12.1)	113 (72.9)	**<0.001**	52.2 (36.2–75.4)
Multiple HPV	30 (0.3)	18 (0.2)	12 (3.4)	**<0.001**	16.8 (8.0–35.2)	2 (1.1)	**0.024**	5.4 (1.2–23.5)	9 (5.8)	**<0.001**	29.8 (13.2–67.5)

Single HPV refers to women infection with only one of HPV genotype; Multiple HPV refers to women infection with at least two kinds of HPV genotype; NILM, negative for intraepithelial lesion or malignancy; ≧ASC-US, include atypical squamous cells of undetermined significance (ASC-US), low-grade squamous intraepithelial lesion (LSIL), atypical squamous cells that cannot exclude HSIL (ASC-H), high-grade squamous intraepithelial lesion (HSIL), atypical glandular cells (AGC). Bold type indicates statistically significant values

*P value, compare the prevalence of HPV genotypes between NILM and abnormal cytological findings using χ2 test or Fisher exact test

** OR (95% CIs), Odd Ratio (95% Confidence Intervals).

### Prevalence of STIs and bacterial vaginosis by cytology status

As shown in **Table 3**, compared to the NILM group, the prevalence of *Chlamydia trachomatis* infection, *Neisseria gonorrhoeae* infection, and bacterial vaginosis were significantly higher in ≥ASC-US group and ASC-US group. In the LSIL+HSIL group, the prevalence of *Chlamydia trachomatis* infection, *Neisseria gonorrhoeae* infection, and Vulvovaginal candidiasis were significantly higher than the NILM group.

**Table 3:** The prevalence of STIs, bacterial vaginosis and social-demographic factors by cytology status.

Variables	Overall (n = 9090)	NILM (n = 8733)	≥ASC−US (n = 357)			ASC−US			LSIL+HSIL		
						(n = 181)			(n = 155)		
	n (%)	n (%)	n (%)	*P* value*	OR (95% CIs)**	n (%)	*P* value*	OR (95% CIs)**	n (%)	*P* value*	OR (95% CIs)**
*Chlamydia trachomatis* (+) ^*a*^	375 (4.1)	346 (4.0)	29 (8.1)	**<0.001**	2.1 (1.4–3.2)	16 (8.8)	**0.001**	2.4 (1.4–4.0)	12 (7.7)	**0.020**	2.0 (1.1–3.7)
*Neisseria gonorrhoeae* (+) ^*b*^	16 (0.2)	12 (0.1)	4 (1.1)	**<0.001**	8.2 (2.6–25.7)	2 (1.1)	**0.006**	8.1 (1.8–36.5)	2 (1.3)	**0.003**	9.5 (2.1–42.8)
*Trichomonas vaginalis* (+) ^*c*^	32 (0.4)	30 (0.3)	2 (0.6)	0.502	1.6 (0.4–6.9)	1 (0.6)	0.640	1.6 (0.2–11.9)	0	—	—
Vulvovaginal candidiasis (+) ^*d*^	529 (5.82)	516 (5.9)	13 (3.6)	0.076	0.6 (0.3–1.1)	8 (4.4)	0.401	0.7 (0.4–1.5)	3 (1.9)	**0.048**	3.2 (1.0–10)
Bacterial Vaginosis (+) ^*e*^	206 (2.3)	190 (2.2)	16 (4.5)	**0.005**	2.1 (1.3–3.6)	11 (6.1)	**0.001**	2.9 (1.6–5.4)	5 (3.2)	0.380	1.5 (0.6–3.7)
Age (40−60) ^*f*^	4857 (53.4)	4643 (53.2)	214 (59.9)	**0.012**	1.3 (1.1–1.6)	108 (59.7)	0.083	1.3 (1–1.8)	90 (58.1)	0.226	1.2 (0.9–1.7)
Contraception (Condom) ^*g*^	5379 (59.2)	5151 (59.0)	228 (63.9)	0.066	1.2 (1.0–1.5)	117 (64.6)	0.126	1.3 (0.9–1.7)	96 (61.9)	0.459	1.1 (0.8–1.6)
APOs (Yes) ^*h*^	1619 (17.8)	1559 (17.9)	60 (16.8)	0.613	0.9 (0.7–1.2)	27 (14.9)	0.308	0.8 (0.5–1.2)	28 (18.0)	0.945	1 (0.7–1.5)
Ethnicity (Minority) ^*i*^	274 (3.0)	266 (3.1)	8 (2.2)	0.385	0.7 (0.4–1.5)	6 (3.3)	0.835	1.1 (0.5–2.5)	1 (0.7)	0.117	0.2 (0–1.5)
Marital status (Married) ^*j*^	9029 (99.3)	8677 (99.4)	352 (98.6)	0.093	0.5 (0.2–1.1)	179 (98.9)	0.448	0.6 (0.1–2.4)	152 (98.1)	0.062	0.3 (0.1–1.1)

^*a b c d e*^ (+) refers to women not only positive for corresponding variables but also may co-infection with other sexually transmitted pathogens as well and/or positive for bacterial vaginosis at the same time

^*f*^ Age (40–60), compared to 20–30 age group

^*g*^ Contraception (condom), compared to those participants who adpoted contraception methods other than condom, including oral pills, intrauterine devices (IUD), injectables, and vaginal ring

^*h*^ APOs, adverse pregnancy outcomes include having any lifelong experience of abortion, premature delivery, ectopic pregnancy or infertility

^*i*^ Ethnicity group (Minority), compared to Han ethnicity

^*j*^ Marital status (Married), compared to single or others status, include single, separated, divorced or widowed status

*P value, compare the prevalence of variables between NILM and abnormal cytological findings using χ2 test or Fisher exact test

**OR (95% CI), Odd Ratio (95% Confidence Intervals). ≧ASC-US, include atypical squamous cells of undetermined significance (ASC-US), low-grade squamous intraepithelial lesion (LSIL), atypical squamous cells that cannot exclude HSIL (ASC-H), high-grade squamous intraepithelial lesion (HSIL), atypical glandular cells (AGC). Bold type indicates statistically significant values.

### Factor associated with abnormal cytological findings

As shown in **Table 4**, after adjusted for potential confounders, compared to the NILM group, high-risk HPV infection (AOR = 20.49, 95% CIs = 16.25–25.83, *P*<0.001), *Neisseria gonorrhoeae* infection (AOR = 5.30, 95% CIs = 1.30–21.51, *P* = 0.020), bacterial vaginosis (AOR = 1.94, 95% CIs = 1.08–3.47, *P* = 0.026) and 40–46 years old (compared to 20–39 years, AOR = 1.32, 95% CIs = 1.03–1.68, *P* = 0.027) significantly increased the risk of ≥ASC-US cytology. Compared to the NILM group, high-risk HPV infection (AOR = 7.66, 95% CIs = 5.45–10.75, *P*<0.001), *Neisseria gonorrhoeae* infection (AOR = 5.72, 95% CIs = 1.12–29.10, *P* = 0.036) and bacterial vaginosis (AOR = 2.64, 95% CIs = 1.38–5.04, *P* = 0.003) significantly increased the risk of ASC-US cytology, and only high-risk HPV infection (AOR = 68.96, 95% CIs = 46.45–102.39, *P*<0.001) significantly increased the risk of LSIL+HSIL cytology.

**Table 4:** Independent factors associated with abnormal cytological findings.

Variables	≥ASC-US		ASC-US		LSIL+HSIL	
	Adjusted OR (95% CIs)*	*P* value**	Adjusted OR (95% CIs)*	*P* value**	Adjusted OR (95% CIs)*	*P* value**
High-risk HPV (+)	20.49 (16.25–25.83)	<0.001	7.66 (5.45–10.75)	<0.001	68.96 (46.45–102.39)	<0.001
*Chlamydia trachomatis* (+)	1.27 (0.81–1.99)	0.300	1.66 (0.96–2.87)	0.071	0.97 (0.49–1.91)	0.927
*Neisseria gonorrhoeae* (+)	5.30 (1.30–21.51)	0.020	5.72 (1.12–29.10)	0.036	3.73 (0.55–25.21)	0.177
Vulvovaginal candidiasis (+)	1.56 (0.86–2.84)	0.146	—	—	2.83 (0.85–9.46)	0.090
Bacterial Vaginosis (+)	1.94 (1.08–3.47)	0.026	2.64 (1.38–5.04)	0.003	—	—
Age (40–60)^*a*^	1.32 (1.03–1.68)	0.027	1.26 (0.91–1.73)	0.162	—	—
Contraception (condom)^*b*^	1.05 (0.82–1.35)	0.705	1.13 (0.81–1.56)	0.474	—	—
Ethnicity group (Minority)^*c*^	—	—	—	—	0.15 (0.02–1.10)	0.061
Marital status (Married)^*d*^	0.68 (0.24–1.92)	0.463	—	—	2.73 (0.50–14.75)	0.244

^*a*^ Age (40–60), compared to 20–30 age group

^*b*^ Contraception (condom), compared to other contraception methods refer to those other than condom, including oral pills, intrauterine devices (IUD), injectables, and vaginal ring

^*c*^ Ethnicity group (Minority), compared to Han ethnicity

^*d*^ Marital status (Married), compared to single or others status, include single, separated, divorced or widowed status; ≧ASC-US, include atypical squamous cells of undetermined significance (ASC-US), low-grade squamous intraepithelial lesion (LSIL), atypical squamous cells that cannot exclude HSIL (ASC-H), high-grade squamous intraepithelial lesion (HSIL), atypical glandular cells (AGC).

*Adjusted OR (95% CIs), Adjusted Odd Ratio (95% Confidence Intervals)

** *P* value, Wald test for AOR value.

## Discussion

In the present study, we investigated the association of STIs and bacterial vaginosis with cervical cytology abnormalities in the general population. Our main findings were women with *Neisseria gonorrhoeae* infection or bacterial vaginosis exhibited an increased risk of ≥ASC-US and ASC-US cytology after adjusted for carcinogenic HPV-positive status, suggesting *Neisseria gonorrhoeae* infection or bacterial vaginosis may act as an independent risk factor for atypical squamous cells formation, predominantly for ASC-US cytology.

In the 2001 Bethesda system, atypical squamous cells does not rule out the diagnosis of abnormal cytology and all atypical squamous cells is considered to be suggestive of squamous intraepithelial lesions (SIL), and atypical squamous cells are qualified as “of undetermined significance” (ASC-US) or “cannot exclude HSIL” (ASC-H) [26]. In the present study, ASC-US cytology (2.0%) was the most prevalent abnormal cytological findings among participants and accounts for ninety-two percent of atypical squamous cells. Although ASC-US cytology was defined as low-grade epithelial cell abnormality [26], about 3.5% of cervical cancers are from the follow-up of ASC-US cytology [27]. ASC-US cytology consists of a wide variety of cervical cytological lesions, such as, CIN-2 or CIN-3 [28], and women with ASC-US are at an increased risk of developing SCC. Therefore, we inferred that *Neisseria gonorrhoeae* infection in genital sites and/or bacterial vaginosis may increase the risk of cervical epithelial cell abnormalities. A previous study concluded that bacterial vaginosis [29] was significantly correlated with the presence of cervical intraepithelial neoplasia such as CIN-1 or CIN-2/3. In line with previous reports, we found that bacterial vaginosis was associated with an increased risk for ASC-US cytology, which implies that evaluation of bacterial vaginosis will be helpful to the management and treatment of patients with abnormal cytology.

In the present study, the prevalence of high-risk HPV in women with abnormal cytology was 52.1%, which is consistent with Northwest China (53.3%) [30] but there was a big gap with Northeast China (62.8%) [31]. In agreement with the previous study [1], women with high-risk HPV infection exhibited a high risk of cervical cytology abnormalities (Odd Ratio values range from 7.66 to 68.96). Meanwhile, we found that the prevalence of HPV genotypes (except for HPV-18, HPV-31, HPV-56, HPV-66) was significantly different between the NILM group and ASC-US group, which means that the surveillance of HPV genotypes is an important tool in the triage of ASC-US cytology [32, 33]. HPV-52 was the most prevalent genotype in the studied population (1.8%), followed by HPV-16 (1.1%), HPV-58 (1.0%), HPV-51 (0.4%), which is consistent with previous reports conducted in the same areas of China [34].

At present, it is unclear whether a single *Chlamydia trachomatis* infection could trigger the formation of cervical lesion, because *Chlamydia trachomatis* is independently associated with persistence of high-risk HPV [35]. Although the prevalence of *Chlamydia trachomatis* in ASC-US group or LSIL+HSIL group was significantly higher than NILM group, a multivariate logistic regression analysis revealed that *Chlamydia trachomatis* infection may not act as an independent risk factor for atypical squamous cells formation after potential confounders controlled, which reinforces the conclusion of Safaeian et al.’s study [18]. Theoretically speaking, *Chlamydia trachomatis* infection triggers the formation of oxidative reactive species (ROS), induces genetic instability, and inhibits damaged DNA repair pathways in the endocervical epithelial cells [36, 37], which may facilitate the entries of HPV and cervical lesions. We deduced that the *Chlamydia trachomatis* infection may aggravate cervical lesions triggered by high-risk HPV infection and play a possible synergistic action with high-risk HPV in cervical lesion progression [9].

Furthermore, management of women with ASC-US cytology confused clinicians in the past decades [28, 38, 39], until recently, a systematic review concluded that HPV DNA test shows higher accuracy and sensitivity than repeat cytology tests in triage women with ASC-US cytology[33]. The association between ASC-US cytology and *Neisseria gonorrhoeae* infection or bacterial vaginosis in our study provided a clue that evaluation of *Neisseria gonorrhoeae* DNA and/or bacterial vaginosis may be a supplementary tool to triage women with ASC-US cytology. Therefore, it is necessary to evaluate the risk of SIL in women with co-presence of ASC-US and *Neisseria gonorrhoeae* infection or bacterial vaginosis in the future study, which may improve our management of women with ASC-US cervical cytology.

The current study has several limitations. Firstly, the methodology used for the identification of *Trichomonas Vaginalis* and Vulvovaginal candidiasis may limit the strengthening of our results. For example, wet mount microscopy of *Trichomonas Vaginalis* has low sensitivity, which ranges from 44% to 68%, though with 100% specificity [40], but microscopic examination of vaginal wet mounts has the advantage of providing instant results as a point-of-care test and is widely available and relatively inexpensive. Therefore, a highly sensitive detection method such as nucleic acid amplification test is needed in the further study to explore the relationship between *Trichomonas Vaginalis* or Vulvovaginal candidiasis and abnormal cytology. Secondly, without identification and characterization of bacterial vaginosis-associated pathogens in the present study limited the analysis of the association between bacterial vaginosis-associated pathogens and abnormal cervical cytology. Consolaro MEL et al.’s [41] study revealed that coinfection between bacterial vaginosis (include Gardnerella vaginalis or Megasphaera type I) with HPV was associated with an increased risk for LSIL or HSIL. Hence, it is necessary to explore the association between bacterial vaginosis-associated pathogens and abnormal cervical cytology after adjusted for carcinogenic HPV-positive status in future studies. Thirdly, because of the standard practice of management for ASC-US cytology is colposcopy or follow-up, information about histological confirmation of the cytological outcomes in ASC-US was missing, which limits the analysis of the association between STIs and histological status.

## Conclusions

In conclusion, this study provided an opportunity to determine the prevalence of STIs and bacterial vaginosis in different cervical cytological findings. We found that *Neisseria gonorrhoeae* infection in genital sites and/or bacterial vaginosis may independently increase the risk for cervical cytology abnormalities, which suggests that performing non-HPV STIs tests will be helpful to diagnosis and treatment of patients in routine cervical cancer screening. Meanwhile, these findings enhance our understanding of the etiology of abnormal cervical cytology, and may also be useful for the management of women with ASC-US cervical cytology.

## Supporting information

S1 File STROBE Statement—checklist of items that should be included in reports of observational studies. (DOCX)

S1 Dataset (XLSX)

## Abbreviations

STIs: Sexually transmitted infections
HPV: human papillomavirus
SCC: Squamous cell cervical carcinoma
CIN: Cervical intraepithelial neoplasia
SIL: Squamous intraepithelial lesion
ASC-US: Atypical squamous cells of undetermined significance
HSIL: high-grade squamous intraepithelial lesion
NILM: Negative for intraepithelial lesion or malignancy
AGC: Atypical glandular cells
CIs: confidence intervals
OR: Odds ratios
AOR: Adjusted odds ratios.
