# Correction: Associations of sexually transmitted infections and bacterial vaginosis with abnormal cervical cytology: A cross-sectional survey with 9090 community women in China

**DOI:** 10.1371/journal.pone.0238272

**Published:** 2020-08-20

**Authors:** Wu Li, Lan-lan Liu, Zhen-zhou Luo, Chun-yan Han, Qiu-hong Wu, Li Zhang, Li-shan Tian, Jun Yuan, Tao Zhang, Zhong-wei Chen, Tu-bao Yang, Tie-jian Feng, Min Zhang, Xiang-sheng Chen

The affiliation for the 12^th^ author is incorrect. The correct affiliation is not indicated. Tie-jian Feng is not affiliated with #1 but with: Shenzhen Center for Disease Control and Prevention, Shenzhen, China.

The affiliation for the 13^th^ author is incorrect. Min Zhang is not affiliated with #1 but with #2: Shenzhen Nanshan Maternity & Child Healthcare Hospital, Shenzhen, China.

## References

[1] Li W, Liu L-l, Luo Z-z, Han C-y, Wu Q-h, Zhang L, et al (2020) Associations of sexually transmitted infections and bacterial vaginosis with abnormal cervical cytology: A cross-sectional survey with 9090 community women in China. PLoS ONE 15(3): e0230712 10.1371/journal.pone.0230712 32214342 PMC7098628

